# Management Approaches and Patient Outcomes for Giant Pituitary Neuroendocrine Tumors Classified as Knosp Grade 3 and 4

**DOI:** 10.7759/cureus.57498

**Published:** 2024-04-03

**Authors:** Kenta Nakase, Fumihiko Nishimura, Shohei Yokoyama, Miho Kakutani, Taekyun Kim, Ryosuke Matsuda, Yasuhiro Takeshima, Shuichi Yamada, Young-Soo Park, Ichiro Nakagawa

**Affiliations:** 1 Department of Neurosurgery, Nara Medical University, Kashihara, JPN

**Keywords:** outcome, complication, multimodal support, giant pituitary neuroendocrine tumor, endoscopic endonasal surgery

## Abstract

Background

Treatment of patients with a giant pituitary neuroendocrine tumor (GPitNET) is challenging. Here, we present the methods used for the clinical management of patients who underwent GPitNET resection mainly via endoscopic endonasal surgery along with multimodal support to avoid surgical complications, which can affect the outcomes.

Methodology

The medical records of 25 patients with a GPitNET who underwent endonasal endoscopic surgery were retrospectively reviewed. Complications were analyzed and factors affecting the extent of resection were evaluated.

Results

Gross total resection was achieved in six (24%), near-total resection (>90%) in nine (36%), and partial resection in 10 (40%) patients. Multivariate analyses revealed that tumors invading the middle fossa had negative effects on the extent of resection (odds ratio = 0.092, p = 0.047). Postoperative vision improved or normalized in 16 (64%), remained stable in eight (32%), and worsened in one (4%), while a new hormonal deficit was noted in seven (28%) patients. Complications included permanent oculomotor nerve palsy in one (4%) and transient oculomotor palsy in one (4%), apoplexy of the residual tumor resulting in ischemic stroke in one (4%), postoperative cerebrospinal fluid leakage in one (4%), and permanent diabetes insipidus in six (24%) patients.

Conclusions

For GPitNETs that extend into the middle fossa, our study underscored the difficulties in surgical extraction and the necessity for tailored treatment approaches. To ensure the safest and most complete removal possible, the surgical strategy must be specifically adapted to each case. Additionally, employing a comprehensive support approach is essential to reduce the chance of complications in patients impacted by this condition.

## Introduction

Treatment of a patient with a giant pituitary neuroendocrine tumor (GPitNET) is challenging [1,2]. It is difficult to remove a tumor with a complicated shape that involves the vascular structure and cranial nerves without causing any damage. The standard goal of treatment for patients with this tumor is optic apparatus decompression and maximal safe resection [3]. Effective surgical management is important to treat patients and lead to good outcomes [4], with endoscopic endonasal surgery (EES) being the primary procedure employed to remove a pituitary neuroendocrine tumor (PitNET) [5-10]. Some GPitNETs extend to the middle fossa and suprasellar regions, making them difficult to remove with only an endonasal approach. It has been reported that a multiple staged operation and/or combination surgery with transcranial and endonasal approaches are necessary for affected patients [4]. It has also been shown that partial resection (PR) of a GPitNET is associated with postoperative apoplexy, with related cases known to have high rates of morbidity and mortality [11]. The complication rate has been reported to be about 10-20% [3-5,11]. Additional therapy is usually necessary to obtain long-term control of tumor growth. The goal of the present study was to evaluate the extent of resection, as well as visual and endocrine outcomes, in patients who underwent resection of a GPitNET, with particular emphasis given to complication avoidance and better management of the surgical approach through multimodal support options.

## Materials and methods

Patient population

From July 2010 to September 2021, 228 patients underwent EES for PitNETs at Nara Medical University, Kashihara, Japan. Among these, the medical records of 25 consecutive patients with GPitNETs (40 mm or greater) were retrospectively examined. Only those patients with prolactinomas who either did not respond to dopamine agonists or experienced significant side effects were considered for surgical treatment. The study received approval from the Ethics Committee of the Nara Medical University of Medical Sciences (approval number: 2652), and every participant provided written informed consent.

Preoperative evaluation

All patients underwent physical and ophthalmological examinations, endocrinological assessments, and MRI scans. Additionally, comprehensive general and neurological histories were collected for each patient. The maximum diameter, shape, characteristics, anatomical location, and extension of the tumor were determined using MRI. The volume of the tumor before surgery was determined using the formula A×B×C/2, where A, B, and C represent the tumor’s maximum dimensions in the axial, coronal, and sagittal planes, respectively [3,12].

Surgical management

All patients underwent EES using multimodal support, including neuronavigation, visual evoked potential (VEP) monitoring, and extraocular movement monitoring, while two underwent a combination transcranial and endonasal approach at the same time, and one underwent EES first, followed by a transcranial procedure performed in a staged manner. The goal of surgery in each case was optic apparatus decompression and maximum possible safe tumor resection. Gross total resection (GTR) was not possible in the majority of cases because of tumor size, shape, and/or invasion.

Evaluation of surgical results

Clinical outcomes after EES were assessed using postoperative visual tests (visual acuity and visual fields), endocrinological studies, and clinical examinations. The extent of resection was determined through an MRI taken at the initial follow-up, typically one month post-surgery. It was categorized into the following three levels: subtotal resection (STR) for less than 90% removal, near-total resection (NTR) for 90% to 99% removal, and GTR for complete removal [3,13]. The extent of resection was assessed subjectively by two authors, KN and FN, according to intraoperative and postoperative findings.

Although optic apparatus decompression is the goal of surgery rather than GTR in most GPitNET cases, GTR rates were calculated in this study of these irregularly extending invasive giant tumors in an attempt to better understand both the advantages and limitations of EES. Tumor size, shape (rounded, dumbbell, multilobular), and extension (anterior, middle, posterior fossa, ventricular system) were examined as independent factors to evaluate their influence on the degree of tumor resection when EES is applied.

Statistical analysis

Patient demographics, clinical presentation, tumor characteristics, surgical approaches, and outcomes were analyzed using descriptive statistics. GTR rates for each of the studied tumor characteristics were compared using logistic regression analyses within the studied variable. P-values <0.05 were considered to indicate statistical significance. All statistical analyses were performed using SPSS for Windows, version 24.0 (IBM Japan, Tokyo, Japan).

## Results

Patient characteristics

This study included 25 patients, comprising 16 (64%) women ranging in age from 35 to 76 years (median = 58.1 years). The tumor was nonfunctioning in 23 patients, while one had an adrenocorticotropic hormone-secreting tumor and one a prolactin-secreting tumor. Seven patients had undergone surgery previously, either microscopic transsphenoidal surgery or a craniotomy, among whom four had undergone multiple surgeries and one with a nonfunctioning PitNET had received additional radiation therapy for the residual tumor (Table 1).

**Table 1:** Patient and tumor characteristics.

Characteristics	Value (%)
Number of patients	25
Age, years	58.12 ± 11.27
Gender	
Female	16 (64)
Male	9 (36)
Surgery	
Transsphenoidal	23 (92)
Transcranial	0
Combined	2 (8)
Tumor size	44.85 ± 5.47
Shape	
Round	4 (16)
Dumbell shaped	4 (16)
Multilobular	17 (68)
Knosp grade	
1	0 (0)
2	0 (0)
3	10 (40)
4	15 (60)
Hormone secretion	
Nonfunctional	23 (92)
Functional	2 (8)
PRLoma	1 (4)
Cushing disease	1 (4)
Previous surgery	
Primary	18 (72)
Recurrent	7 (28)
Extension	
Anterior fossa	5 (20)
Middle fossa	14 (56)
Posterior fossa	0 (0)
Sphenoid sinus	15 (60)
Suprasellar region	24 (96)
Intraoperative tumor consistency	
Soft	13 (52)
Firm or fibrotic components	12 (48)
Ki-67	
<1%	14 (56)
1–3%	8 (32)
>3%	2 (8)

Clinical presentation

The dominant clinical symptoms at presentation were headache, noted in 24 (96%) patients, and visual impairment affecting visual acuity, the visual field, or both, noted in 22 (88%) patients. Partial or complete pituitary insufficiency was evident in 10 (40%) patients, including hypoadrenalism in eight (32%), hypothyroidism in six (24%), hypogonadism in four males (16%), and growth hormone deficits in 18 (72%). Diabetes insipidus (DI), altered mental status, or hydrocephalus was not evident in any of the patients at presentation, while three (12%) were affected by apoplexy, and two (8%) suffered from cranial nerve palsy (Table 2).

**Table 2:** Clinical presentation. CN = cranial nerve; DI = diabetes insipidus

Clinical presentation	Number of patients (%)
Headache	24 (96)
Visual impairment	22 (88)
Pituitary insufficiency	11 (44)
Apoplexy	3 (12)
CN palsy	2 (8)
Hydrocephalus	0 (0)
Altered mental status	0 (0)
DI	0 (0)

Tumor characteristics

The average GPitNET volume was 23.3 cm^3^ (range = 6.9-67.4 cm^3^), with a rounded shape in four (16.0%), dumbbell shape in four (16.0%), and multilobular form in 17 (68.0%) cases (Table 1).

The tumors in all 25 (100%) cases occupied the sella region, extended to the suprasellar region in 24 (96%), and to the sphenoid sinus in 15 (60%). Using the Knosp criteria, none of the cases was Grade 1 or 2, while 10 (40%) were Grade 3, and 15 (60%) were Grade 4. Grade 4 tumors were found in cases with radiographic evidence of carotid artery involvement. Further intracranial tumor extension into the sagittal plane (anterior fossa), coronal plane (middle fossa), axial plane (posterior fossa), and ventricular system was also assessed, with homogeneous enhancement after contrast administration noted in eight (32%) and heterogeneous enhancement in 15 (60%) cases (Table 1).

Adjuvant therapy

**Figure 1:**
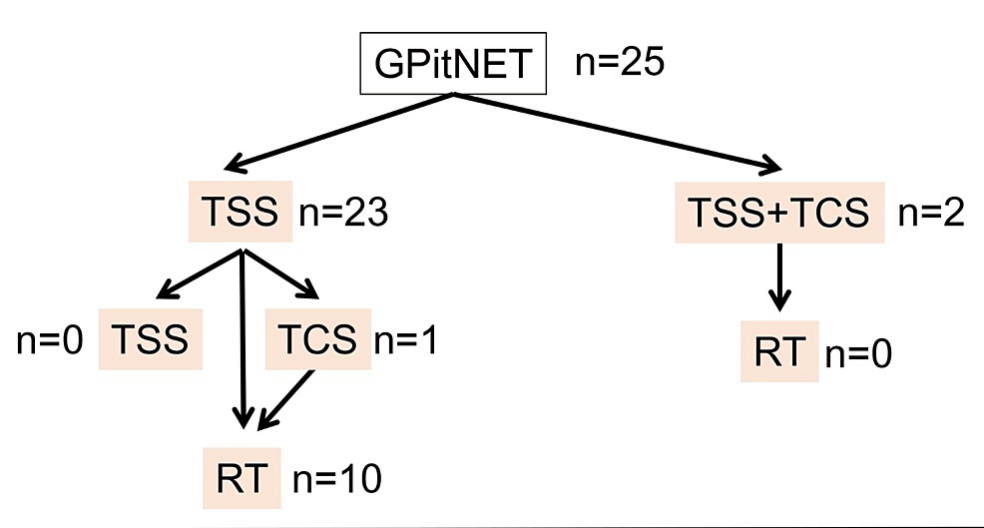
Selection of surgical approach and adjuvant therapy. TSS = transsphenoidal surgery; TCS = transcranial surgery; RT = radiation therapy

Ten patients underwent radiosurgery at a later stage for tumor recurrence or regrowth. One patient with a prolactinoma continued to receive medical treatment following surgery (Figure 1).

Clinical outcomes

Of the 22 patients with visual disturbance, improvement or normalization of vision was achieved in 13 (59%), while a stable condition was noted in eight (36%). One (4.5%) patient experienced visual deterioration due to apoplexy of the residual tumor. All three patients with normal vision before had stable visual function remaining after surgery. As for pituitary function, new hypopituitarism occurred in seven (28%) patients after surgery. In all patients, preexisting cranial nerve palsy improved or normalized after the procedure. One patient had newly developed permanent oculomotor palsy after surgery and another newly transient oculomotor paresis, which resolved within three months. Permanent DI after surgery was noted in six (24%) patients. Furthermore, delayed cerebrospinal fluid (CSF) leakage that required a surgical procedure for repair occurred in one (4%) patient (Table 3). The mean duration of follow-up was 74.1 months. Tumor recurrence/progression occurred in 10 (40%) patients.

**Table 3:** Clinical outcomes. eTSS = endoscopic endonasal transsphenoidal surgery; DI = diabetes insipidus; CSF = cerebrospinal fluid

Clinical outcomes	Number of patients (%)
Outcome in patients with visual impairment before surgery	
Improved	13 (59)
Stable	8 (36)
Worse	1 (4.5)
Outcome in patients with normal vision before surgery	
Stable	3 (100)
Worse	0 (0)
Oculomotor palsy	
Permanent	1 (4)
Transient	1 (4)
Postoperative bleeding (eTSS)	1 (4)
New hypopituitarism	7 (28)
Permanent DI	6 (24)
Delayed CSF leakage	1 (4)

Degree of tumor resection

GTR was achieved in six (24%) patients, NTR (>90%) in nine (36%), and PR in 10 (40%). Factors with influence on achieving GTR were also analyzed. Univariate analysis revealed cavernous sinus invasion (Knosp 4) and tumors extending to middle fossa factors blocking GTR achievement (p = 0.015 and 0.047, respectively). Furthermore, multivariate analyses showed that tumors invading the middle fossa had negative effects on the extent of resection (odds ratio = 0.092, p = 0.047) (Table 4).

**Table 4:** Factors related to achieving GTR. GTR = gross total resection; OR = odds ratio; CI = confidence interval

			Univariate		Multivariate	
Variable	GTR	Non-GTR	OR (95% CI)	P-value	OR (95% CI)	P-value
Shape						
Round	2	2	1.000 (ref.)		－	－
Dumbell	2	2	1.000 (0.063, 15.988)	0.999	－	－
Multilobular	2	15	0.133 (0.011, 1.550)	0.107	－	－
Knosp						
Grade 3	6	4	1.000 (ref.)		－	－
Grade 4	0	15	0.022 (0.001, 0.477)	0.015	－	－
Extension						
Anterior fossa	2	3	2.667 (0.327, 21.733)	0.360	－	－
Middle fossa	1	13	0.092 (0.009, 0.973)	0.047	0.092 (0.009, 0.973)	0.047
Sphenoid sinus	2	13	0.231 (0.033, 1.628)	0.141	－	－
Suprasellar region	18	6	1.054 (0.038, 29.246)	0.975	－	－
Firm tumor	1	11	0.145 (0.014, 1.498)	0.105	－	－

Representative case

**Figure 2:**
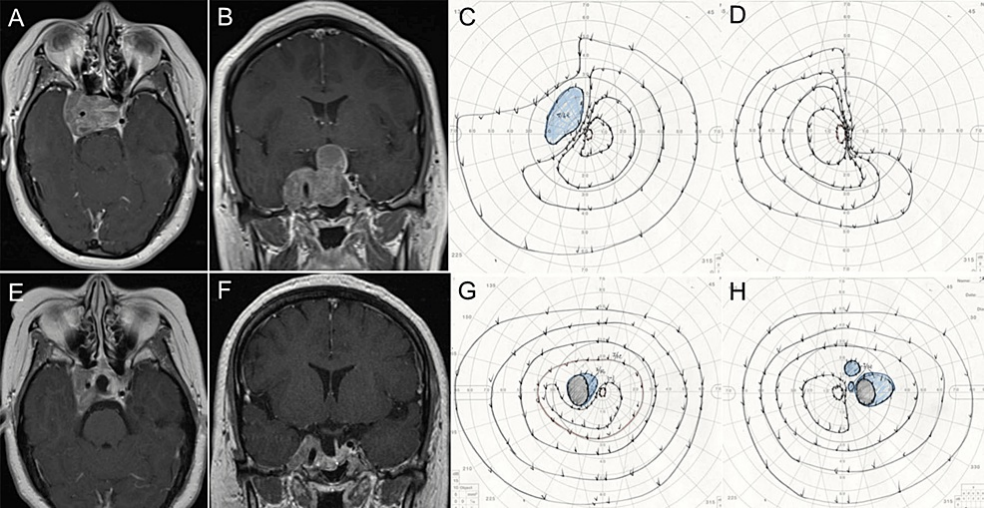
MRI images and visual field test obtained in a 35-year-old woman presenting with right oculomotor palsy and visual field loss. (A, B) Axial and coronal MRI shows a giant intra- and suprasellar tumor extending to the middle fossa (Knosp grade 4). (C, D) Preoperative visual field test shows defects in the upper temporal regions of both eyes. (E, F) Axial and coronal MRI postoperatively shows a residual tumor in the cavernous sinus. (G, H) Preoperative visual field test shows the improvement of visual field defects.

A 35-year-old woman was examined by our department due to a right oculomotor nerve palsy and visual field loss. MRI revealed a giant intra- and suprasellar tumor extending to the middle fossa (Figures 2A, 2B). A visual field examination indicated defects in the upper temporal regions of both eyes (Figures 2C, 2D). EES was performed with neuronavigation, motor-evoked potential monitoring, VEP monitoring, and extraocular movement monitoring. MRI scans post-surgery indicated a partial tumor resection (Figures 2E, 2F). The patient’s oculomotor nerve palsy and visual field deficits showed improvement (Figures 2G, 2H). Gamma knife radiosurgery (15 Gy) for residual tumor was performed seven months after the surgery. There has been no tumor recurrence five years after the surgery.

## Discussion

Treatment for a GPitNET is challenging because of size, invasiveness, and extrasellar extension [13]. These are linked to a lower likelihood of achieving GTR, leading to higher rates of recurrence, as well as elevated risks of postoperative complications and mortality, culminating in a generally unfavorable long-term outlook [14]. Especially for GPitNETs with middle fossa extension, this study indicated the difficulty of resection and the necessity of tailored management.

Previous reports showed cavernous sinus invasion is one of the main limitations that precludes complete tumor resection [8,9,15-17]. Univariate analysis of the present cases revealed that cavernous sinus invasion (Knosp 4) and tumor extension to the middle fossa led to GTR (p = 0.015 and 0.047, respectively), while multivariate analysis revealed that tumors invading the middle fossa had negative effects on the extent of resection (OR = 0.092, p = 0.047) (Table 4). Similar to the current study, Koutourousiou et al. identified that the spread to the middle fossa significantly hinders the extent of resection, with a notable p-value of 0.045 [3]. Additionally, Ceylan et al. underscored the criticality of recognizing the heightened risk of tumor remnants in areas extending beyond a 10 mm margin around the anterior curve of the cavernous segment of the carotid artery during endoscopic surgeries [18]. Extensions into the intraventricular or posterior fossa regions were found not to affect surgical outcomes, aligning with previous reports.

Previous studies have also highlighted the relationship between the multilobulated shape of tumors and a reduced GTR rate, as documented by previous reports [3,19]. The existence of multiple compartments is deemed a significant challenge in achieving complete resection, often due to tumor spread into the subarachnoid space and the entanglement of arteries. In our study, the multilobular configuration showed a tendency toward non-GTR, although this association was not statistically significant (p = 0.107).

The risk of critical postoperative bleeding is heightened for these tumors due to the possibility of incomplete resection [20]. Thus, the primary objective of surgery for GPitNET, especially for these tumors, is to achieve a maximal safe resection, resulting in decompression of the optic apparatus to gain visual improvement, and recovery from endocrinological and neurological symptoms [3]. To achieve this goal, the surgical approach must be tailored for individual cases and multimodal support should be applied to prevent complications in affected patients [8].

The surgical approach should be tailored for individual cases according to GPitNET size and extension, its configuration, and the need for hormonal treatment, as well as in consideration of the goal of treatment specified by the patient [4]. EES is the most commonly used for GPitNET removal. In the present series of 25 patients, EES was used for 23 (92%), while two (8%) underwent a combination of EES and TCA due to the larger size of the tumor and more invasion into the middle fossa, respectively.

In our patients, neuronavigation, Doppler ultrasound, and neuromonitoring were applied as multimodal support. Neuronavigation is useful to recognize anatomical orientation and helps avoid injury to vital structures, while Doppler ultrasound is also helpful in detecting the location of the carotid artery. Furthermore, neuromonitoring is a valuable tool to monitor and maintain neurofunction, especially regarding visual function by VEP monitoring [21]. Motor evoked function is helpful to monitor motor function, and extraocular movement monitoring is also important to monitor oculomotor nerve and abducent nerve function when a GPitNET shows cavernous sinus invasion [22,23].

Regarding the degree of tumor resection, our study showed relatively low GTR in six (24%) patients compared to previous reports [13]. These results may occur because our study included many patients with Knosp grade 4 (n = 15, 60%), which is associated with the limitation in achieving GTR. Moreover, in this study, there tend to be many multilobular types (n = 17, 64%). This trend toward prioritizing maximum safe resection over GTR is believed to be the reason behind these findings.

Although postoperative visual improvement in the present series was seen in 13 (59%) patients, which is relatively low compared to previous reports, 21 (95%) patients showed either improved or stable postoperative visual function, which is considered to be good results [1]. Unfortunately, one showed a worsened visual outcome because of postoperative bleeding from the residual tumor.

The rate of complications for GPitNET cases has been reported to range from about 10% to 20%, generally because of the large size, invasiveness, and irregular extension [3,5,24,25]. There are several possible life-threatening complications, such as carotid injury, stroke, postoperative pituitary apoplexy, and meningitis. To avoid these complications, the primary surgeon should focus carefully on dissecting the tumor from the neurovascular structure and employ multimodal support methods for safety, such as navigation, monitoring, and Doppler ultrasound. To keep perforators safe, tumors with vessel involvement may sometimes be intentionally left to avoid injury or vasospasm. In this series of cases, none had carotid injury, while one (4%) showed oculomotor palsy, one (4%) experienced delayed CSF leakage, and six (24%) had newly occurring DI, results comparable to previous reports [2,13,26].

As the findings presented here are from a retrospective analysis, a small surgery cohort, and the lack of a control group, the power of the statistical analyses may be limited. A larger multicentric study is mandatory for external validation and confirmation of the clinical and surgical relevance of the present findings.

## Conclusions

In cases of GPitNET extending into the middle fossa, our research highlighted the challenges associated with surgical removal and the importance of personalized treatment strategies. Achieving a maximal safe resection requires that the surgical plan be customized for each case and that multimodal support be implemented to minimize the risk of complications in affected patients.
